# Virtual reality as a non-medical tool in the treatment of anxiety, pain, and perception of time in children in the maintenance phase of acute lymphoblastic leukemia treatment

**DOI:** 10.3389/fonc.2024.1303421

**Published:** 2024-03-19

**Authors:** Liliana Velasco-Hidalgo, Alejandro González-Garay, Blanca Angélica Segura-Pacheco, Ana Luisa Esparza-Silva, Miguel Enrique Cuéllar Mendoza, Cecilia Ochoa-Drucker, Sofía Campos-Ugalde, Luis Eduardo Bernabé-Gaspar, Marta Zapata-Tarrés

**Affiliations:** ^1^Oncology Department, Instituto Nacional de Pediatría, Mexico City, Mexico; ^2^Methodology Research Department, Instituto Nacional de Pediatría, Mexico City, Mexico; ^3^Research Department, Triovance, Mexico City, Mexico; ^4^Research Coordination, Fundación Instituto Mexicano del Seguro Social (IMSS A.C.), Mexico City, Mexico; ^5^Biochemestry Department at Universidad Nacional Autónoma de México (UNAM), Mexico City, Mexico; ^6^Sohma Psyco-Oncology Group, Mexico City, Mexico

**Keywords:** acute lymphoblastic leukemia, virtual reality, pediatric oncology, therapeutics, Mexico

## Abstract

**Introduction:**

Management of pediatric cancer patients involves invasive procedures such as punctures, injections, catheter placements, and chemotherapy which can generate fatigue, nausea, vomiting, anxiety, and pain. Virtual Reality (VR) is a nonpharmacological intervention classified as a cognitive-behavioral method to relieve symptoms.

**Methods:**

We designed a crossover protocol and included 20 patients between 9 and 12 years old; ten were male. All patients had acute lymphoblastic leukemia diagnosis and were treatedwith St. Jude’s XV protocol in the maintenance phase. Pain and anxiety were measured with validated scales in the pediatric population.

**Results:**

Although we used a small group of patients, we found statistical difference in the reduction of anxiety and perception of time.

**Discussion:**

These results open a window to non-pharmacological treatments and show a strategy to improve quality of life in children inside the hospital.

## 1 Introduction

In Mexico, cancer is the second most common cause of death in the pediatric population. We estimate that between 6000 and 7000 new cases are diagnosed per year (1, 2). Acute lymphoblastic leukemia is the most frequent cause of cancer in Mexico and around the world. During the diagnostic and therapeutic period, patients can be prescribed more than 10 drugs and undergo different painful procedures and hospitalization.

The symptoms associated with diagnosis, treatment, and rehabilitation of children with cancer such as fatigue, nausea, vomiting, anxiety, and pain affect their quality of life and the quality of life of their relatives. They also contribute to diminished adherence to treatment (3).

Virtual reality (VR) can be defined as a three-dimensional spatial environment generated by a computer with real-time participation. This can be achieved including three characteristics: immersion, presence, and interactivity (4). Because VR combines all three sensory modalities, this intervention is ideal for distraction as it captures the attention of various sensory modalities (visual, auditory, and kinesthetic) and diverts attention from the real world, including painful or discomforting stimuli (5). The development of VR has accelerated in recent years, with medical devices being one of the largest growing areas (6).

Virtual reality (VR) was defined in 2002 by Weiderhold and Rizzo as an advanced interface, defining interface as a functional connection between two systems (7, 8), for example between a computer and the computer user. This advanced interface allows the user to interact and feel immersed in a computer-generated environment. VR also allows individuals to hear and feel stimuli that are projected on the VR headsets and gadgets. It is considered immersive because it involves several senses at the same time, does not require previous training, and the headset prevents the user from experiencing external stimuli that compete with what they are perceiving (9, 10).

In Oncology, several protocols using VR as a distraction tool have been explored. In 1999, Schneider conducted a study on 11 children with leukemia or Hodgkin lymphoma, using a crossover model (11, 12). In 2007, Schneider used VR in the relief of symptoms such as anxiety, fatigue, and changes in the perception of time for adult cancer patients. The study also evaluated anxiety and fatigue through questionnaires such as the Revised Piper Fatigue Scale and State Anxiety Inventory (11–13). Because patients only received intervention with VR, no significant difference was found, although the study did observe an effect in the decrease in the perception of time. It was suggested to increase the number of interventions to influence the reduction of fatigue and anxiety.

VR has also been used for pediatric patients. The objective of these studies has been to evaluate the effect of VR in reducing chemotherapy-associated symptoms such as anxiety, pain, nausea, and fatigue immediately after treatment and 48 hours later. The measurement was carried out on three occasions (first event: control without VR; second event: with VR; third event; without VR) using the symptom distress scale (SDS) and State Trait Anxiety Inventory for Children (STAIC-1). The results showed a decrease in the distress symptom scale at the end of chemotherapy, however there was no effect after treatment (13, 14).

The perception of time during a chemotherapy session is an important aspect of the quality of life in patients. In a habitual environment, time is perceived by the comparison between operative memory (stores information for a short time) and a reference memory (stores information for longer time). Unpleasant stimuli cause a distortion in time perception, generally registering an increase of time perception. Introducing a distraction that diverts attention from the unpleasant stimulus means that the perception of time is quicker. In the study by Schneider, Kisby, and Flint, perception of time measurement began from the beginning of the infusion until the end of it and patients were asked about their perception of time. Results show that VR decreases perception of time (14).

Pain produced by cancer, the treatment, or medical procedures can alter patient’s quality of life. The visual analog scale to measure pain has been validated internationally (15, 16). In parallel, anxiety is a transitory state, normally due to a stressful situation, although it can be a personality trait (17). STAIC is a tool designed by Spielberg specifically to measure anxiety; it offers two evaluations: transitory anxiety and anxiety as a personality trait. The scale goes from A to E and includes 20 elements in which the person can indicate how they feel. For the scale A to E, answering options are “nothing”, “Some”, or “A lot”. The scale is used for patients between 9 and 15 years old and has been validated in English and Spanish (18).

Acute lymphoblastic leukemia is the most common malignant neoplasm in pediatrics (19). Survival can reach almost 90% in low-risk cases. Internationally, patients are treated with chemotherapy protocols (20). In Mexico, most of the patients receive St Jude’s XV, which consists of three phases: induction, consolidation, and maintenance (21). Patients in the maintenance phase are clinically stable. This phase last 120 weeks and is a long period for adherence to treatment. To achieve adherence, we must consider the type of disease, the type of treatment, and the management of side effects. The first two factors cannot be modified, but the side effects like nausea, pain, and anxiety can be treated with drugs. The side effects can also be ameliorated with psychological support with nonpharmacological treatments such as VR. VR has not been previously used in Mexico as a non-pharmacological therapy to reduce anxiety and pain during chemotherapy in children.

Our aim was to determine the effectiveness of virtual reality in reducing anxiety and pain in patients between 9 and 12 years old in the maintenance phase of acute lymphoblastic leukemia during the administration of chemotherapy.

## 2 Patients and methods

We designed a crossover protocol. We included 20 patients. The selection criteria were:

Inclusion criteria: Children between 9 and 12 years old in first remission of ALL who speak Spanish treated at the National Institute of Pediatrics in Mexico City from September 2018 to December 2018.

Exclusion criteria: Patients who could not answer the questionnaire, with convulsive crisis, visual or auditive deficiencies, a history of anxiety, or neurological problems.

Elimination criteria: Patients who died during the study development or had convulsive crisis or auditive or visual deficiencies after the study started were analyzed until the event.

We measured the following variables: age, sex, geographic origin, level of study of the patient and parents, risk of ALL, type of chemotherapy, week of treatment, date of chemotherapy application, time of beginning and ending of chemotherapy, pain visual scale before and after chemotherapy application, and anxiety scales before and after chemotherapy application.

All patients had a diagnosis of acute lymphoblastic leukemia and were in remission. All cases were classified and treated with St. Jude’s XV protocol in the maintenance phase. The maintenance phase is characterized by a weekly treatment. All the drugs are intravenous and injected in the ambulatory area of the Oncology Department. The patients are supervised by pediatric oncological nurses and a pediatric oncologist during the morning shift from Monday to Friday.

The consent and assent process were done in the outpatient clinic. In the first week of maintenance the protocol was implemented and patients received VR. Patients received VR with Oculus Go headsets with four videos. The weight of the visors was 468g with no cables. They did not need to be connected to a computer or a telephone. The audio system was included in the headset. Surfaces were smooth and could be easily disinfected.

All patients signed a consent letter for research. The protocol was approved by the ethics and research committee of the National Institute of Pediatrics (Approval number 2018-068/Approval date: November 29^th^, 2018). Twenty patients were included by convenience sampling.

Intervention: before and after the infusion of chemotherapy, the anxiety and pain questionnaires were applied. At the end of the infusion, we questioned the patients about their perception of time. We carried out the first two sessions of chemotherapy without the use of VR. From the fifth to the eighth chemotherapy session, VR was used. In each session the patient used the viewers for 30 minutes. Videos were changed in each session and were in the same order for all the patients. The content of the videos was consulted by a teacher and reviewed by a psychologist. Real time as well as the registration of the time perceived by the patient was recorded by the researcher. Each week, the patient arrived with their companion and all the treatment information was verified. After this, the researcher would go with the patient and apply the before questionnaire, then the patient received VR and, when the chemotherapy was finished, the after questionnaire was applied. All the questionnaires were self-conducted and were done in the same room as the ambulatory oncological area. Patients are with other pediatric oncologic patients in the same area and with one of their parents and a television. The patients included in the protocol had the same conditions as those not included in it. Pain and anxiety were measured with the STAIC questionnaire. We made eight measures. In all the events, we applied the questionnaire before and after the administration of chemotherapy. Although adverse events were not expected, emphasis was placed on the patient’s comfort. If the visor was not comfortable, it was removed. Patients wore disposable masks to avoid any infection from wearing the visor.

The contents of the videos were:

1. Serengeti Tour: the patients “go” on board the Masai vehicle seeing up to 500 different species from the Serengeti reserve.
2. Become a footballer: Patients “live” the training of professional soccer players of two professional Mexican teams.
3. Fun on the go: Patients “ride” a roller coaster.
4. Search and find in Art: Patients appreciate the work of different painters.

Descriptive analysis was carried out using measures of central tendency and dispersion. For quantitative variables, mean with standard deviation or median with minimum-maximum was obtained, while for qualitative variables, frequencies and proportions were calculated due to the distribution of their data with the Kolmogorov-Smirnov normality test. To identify the differences in the STAIC questionnaire score for anxiety and pain, before and after the VR application each week, the Mann-Whitney U test was performed using a significance value of 0.05.

Subsequently, regressions were performed for longitudinal data using fixed effects during the eight weeks of chemotherapy treatment in order to identify significant differences with the use of VR in the STAIC questionnaire score for anxiety and pain.

## 3 Results

We included twenty patients. Ten were male. Eight patients were in primary school, nine in secondary, and three in high school. All patients were from the metropolitan area, all parents were literate, and 13 patients had a high-risk leukemia. All patients were treated with St. Jude’s XV protocol, and all were in the maintenance phase (from week 20 to 45). As treatment is weekly, patients had their appointment every week. Metotrexate, cytarabine, steroids, and cyclophosphamide were the main chemotherapeutics.

The first data analysis we made was the comparison of STAIC questionnaire measures before and after chemotherapy infusion. The analysis considered was regression of fixed effects for longitudinal data. We reported eight weeks, with values before and after the infusion of chemotherapy. Measures were of anxiety state, anxiety status, and pain. In week 1, the average score of the questionnaire was 27 in the anxiety scale with a range between 21 and 43. The average score was 29.5 in the anxiety feature with a range between 21 and 46, and 1 in pain between 0 and 8. From week 1 to week 8 the variability was minimum and, when comparing before and after, we only achieved a significant difference on week 5. (**Tables 1**–**3**).

**Table 1:** Comparison of STAIC questionnaire measures before and after chemotherapy infusion – National Institute of Pediatrics – September to December 2018.

	Variable	Pre-chemotherapy Median (min – max)	Post-chemotherapy median (min – max)	P
**Week 1**	Anxiety state	27 (21 to 43)	27.5 (21 to 46)	0.75
	Anxiety feature	29.5 (25 to 50)	32.5 (23 to 50)	0.20
	Pain	1 (0 to 8)	2 (0 to 9)	0.30
**week 2**	Anxiety state	26 (20 to 43)	28.5 (21 to 50)	0.25
	Anxiety feature	31 (22 to 45)	33.5 (21 to 48)	0.14
	Pain	2 (0 to 8)	2 (0 to 6)	0.14
**week 5**	Anxiety state	28 (20 to 43)	23 (20 to 40)	0.04*
	Anxiety feature	30.5 (21 to 48)	31 (22 to 42)	0.73
	Pain	1 (0 to 8)	1 (0 to 6)	0.73
**week 6**	Anxiety state	27 (20 to 35)	24.5 (20 to 44)	0.28
	Anxiety feature	31 (22 to 42)	31.5 (21 to 44)	0.72
	Pain	1.5 (0 to 2)	1.5 (0 to 4)	1.00
**week 7**	Anxiety state	24.5 (20 to 39)	24.5 (21 to 42)	1.00
	Anxiety feature	30 (24 to 43)	31 (3 to 42)	0.70
	Pain	1.5 (0 to 2)	1 (0 to 2)	0.44
**week 8**	Anxiety state	23 (20 to 45)	24.5 (20 to 50)	0.40
	Anxiety feature	32 (22 to 47)	32 (22 to 46)	1.00
	Pain	1 (0 to 2)	1.5 (0 to 4)	1.00

*Regression of fixed effects for longitudinal data.

**Table 2:** Time perceived (in minutes) – National Institute of Pediatrics – September to December 2018.

Week	Time perceived Median (min – max)	Real time Median (min – max)	P
**Week 1**	30 (1 to 180)	87.5 (1 to 210)	0.08
**Week 2**	40 (2 to 780)	120 (5 to 1440)	0.61
**Week 5**	20 (3 to 600)	40 (10 to 1440)	0.006*
**Week 6**	30 (8 to 960)	47.5 (25 to 1440)	0.28
**Week 7**	25 (2 to 1080)	32.5 (17 to 1440)	0.24
**Week 8**	22.5 (2 to 40)	35 (5 to 60)	0.20

Statistical test = Mann Whitney U test.* It shows statistical significance.

Table 3

**Table:** Comparison of the anxiety state, anxiety feature, and pain with and without VR– National Institute of Pediatrics – September to December 2018.

Treatment anxiety state	Coefficient	IC95%	p
**with VR**	27.95	26.62 to 29.29	0.000*
**without VR**	27.38	26.04 to 28.73	0.000*

*Dynamic panel by fixed effects < 0.05 R^2^ = 0.94, p model = 0.000.

**Table:** 

Treatment anxiety feature	Coefficient	IC95%	P
**with RV**	32.37	31.00 to 33.74	0.000*
**without VR**	32.5	31.11 to 33.88	0.000*

*Dynamic panel by fixed effects < 0.05 R^2^ = 0.95, P model = 0.000.

**Table:** 

Treatment Pain	Coefficient	IC95%	p
**with VR**	1.77	1.47 to 2.07	0.000*
**without VR**	2	1.69 to 2.30	0.000*

*Dynamic panel by fixed effects < 0.05 R^2^ = 0.94, P model = 0.000.

In **Table 2**, we observe the differences between the real time and the time perceived by the patient. In week 1, the time perceived by the patient was on average 30 minutes contrasted with the real time registered which was 87.5 minutes. In week 2, the values were 40 minutes compared to 120 minutes; in week 5, it was20 minutes compared to 40 minutes. In week 1 as well in week 5 we observe statistical differences.

**Figure 1:**
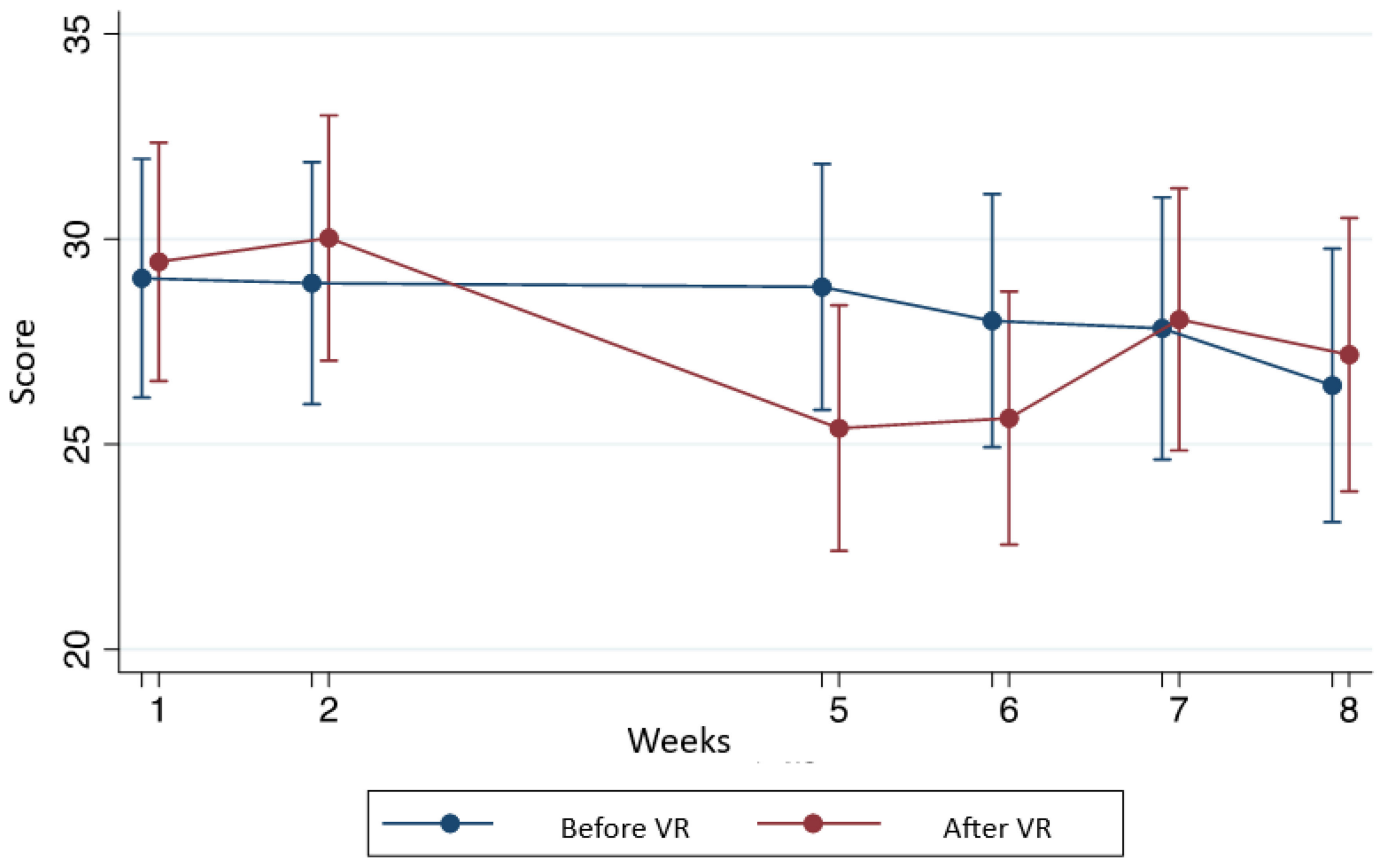
Comparison of the anxiety state before and after the use of VR according to the STAIC questionnaire.

**Figure 2:**
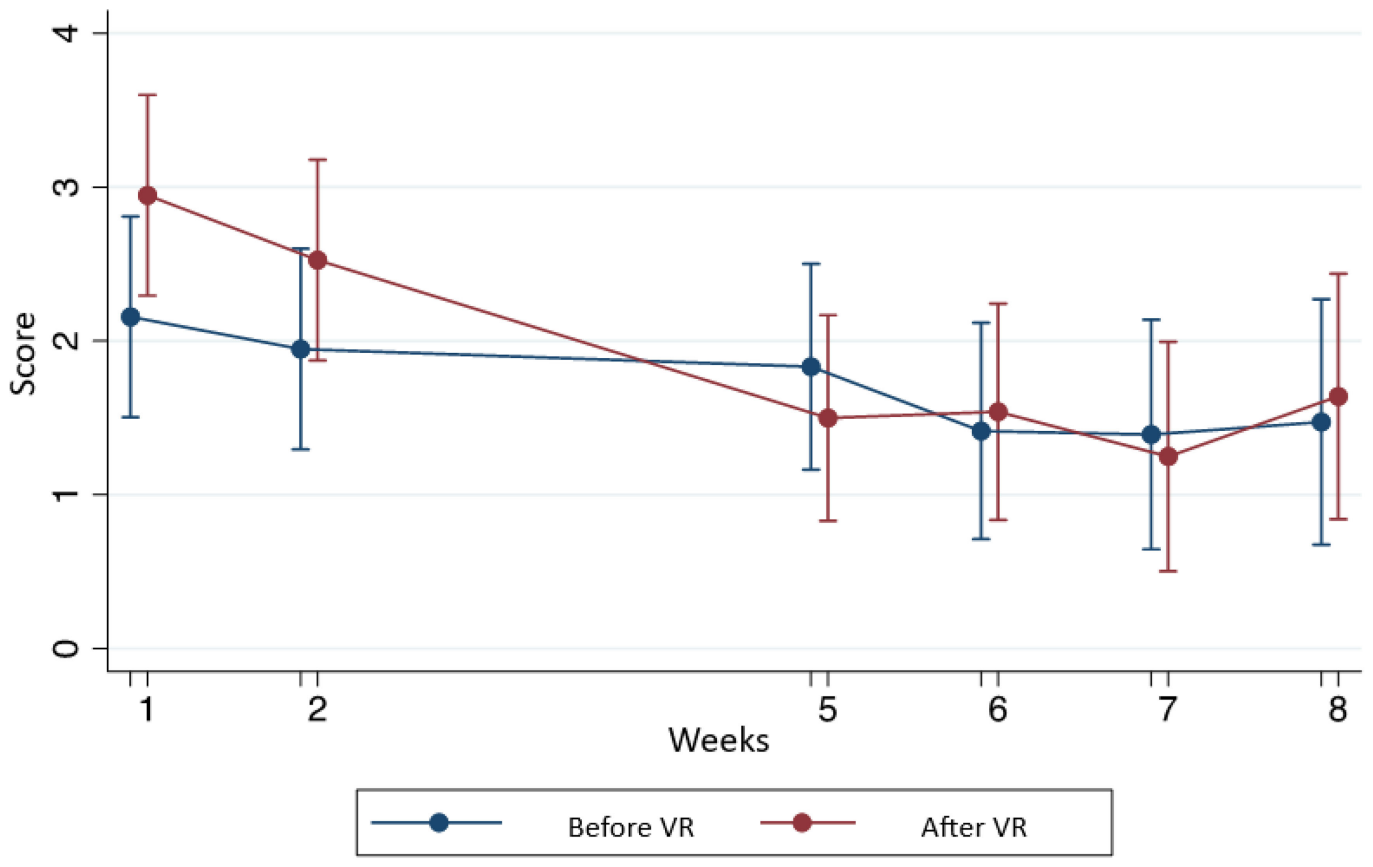
Comparison of pain before and after the use of VR according to the STAIC questionnaire.

**Figure 3:**
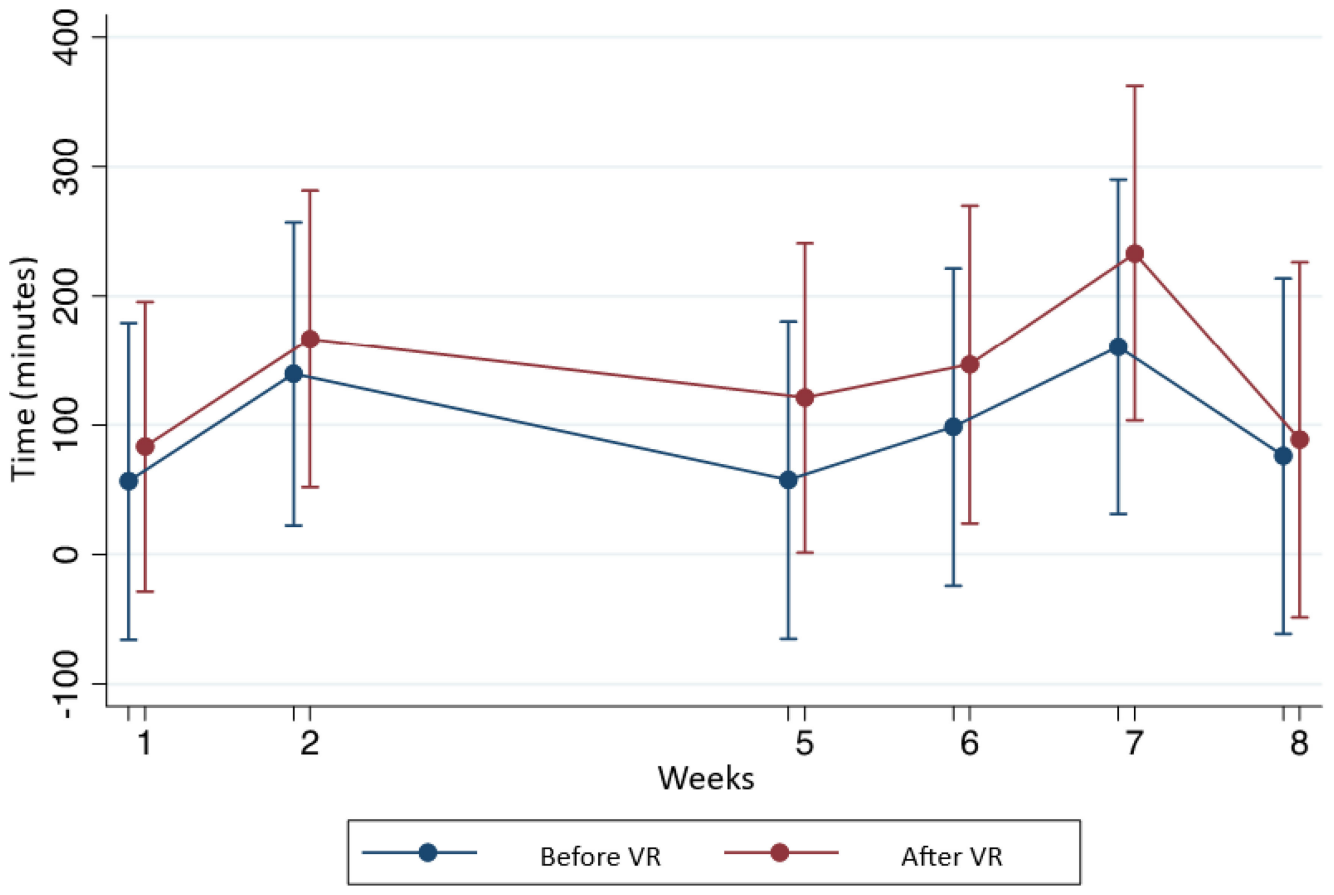
Time perceived during chemotherapy.

**Table 3** show statistical differences with and without VR in the score in anxiety state, anxiety feature, and pain. **Figures 1**-**3** shows this comparison.

We did not register any adverse events. Patients accepted and enjoyed the VR beyond the psychological tests.

## 4 Discussion

Higher anxiety levels experienced by children before and during medical procedures are associated with peri- and post-procedural pain and can lead to inadequate relief of pain and distress. The use of VR as a distraction helps to reduce anxiety and pain and is also linked to greater pain tolerance (22). In a study conducted in Australia and New Zealand by Evelyn Chan et al., with children aged 4-11 years undergoing intravenous cannulation, which is an invasive medical procedure, the use of VR proved to be efficacious in decreasing pain (23). Another study performed by Gerçeker GÖ, et al. found that VR distraction decreased the fear and anxiety feelings and also reduced the pain score results before and after the procedure of port needle insertions for Pediatric Hematology-Oncology patients aged 6-17 years (24). The selection criteria in age, disease, and state of the disease were strict so the study has a very high internal validity but low external validity. At this point, we consider that these characteristics give more power to the results and facilitate the creation of a new line of research.

The most interesting result considering a pediatric point of view is the reduction of perceived time of chemotherapy administration, as well as the reduction of anxiety and pain.

Analyzing results week by week, we observe that the main differences are registered in the first two weeks, week 5 and 6. This can be explained considering that week 1 is the first time the patients are exposed to the questionnaire and in week 5 it is the first time they are exposed to VR.

Analyzing the time perception, we observe the most important difference in week 5. This can be easily explained due to the use of VR. Current management of acute pain include opioids and physical exercise or therapy, but these interventions are inadequate. The current application of VR therapy demonstrates the potential to redefine a novel approach to treat acute pain in the clinical setting, because VR not only provides a distraction from pain stimuli but also a decrease of time perception (25). However, we observed a statistical difference in week 1 and in the first week we did not make an intervention. This difference suggests that patients usually perceive time in a different way or cannot measure time objectively. There is no information about the time perceived by patients in an ordinary setting. In our study we can assume that patients perceive time to be much longer than real time when chemotherapy is administrated. It is reported in literature that there is a close relation between a person’s current emotional status and their perception of time: entertainment activities appear to accelerate time flow, while the monotony of uneventful circumstances seems to instinctively decelerate time. This is an important topic for discussion in future research.

Although we included a small group of patients, we found statistical difference in anxiety. Although we made no difference in gender or age in the inclusion criteria, it is important to know that adolescents have a worse experience with the disease because of social changes. The adolescent population represents an important percentage of patients with cancer diagnosis, so in this specific population, it is necessary to implement novel strategies to decrease pain and anxiety associated with chemotherapy administration (24). The use of new technologies can help mitigate symptoms such as fatigue or depression. These types of interventions using VR before medical procedures will help reduce the fear associated with medical interventions and medical procedures, will increase pain tolerance, decrease healthcare-related anxiety levels, and help to prevent healthcare avoidance in adulthood (26).

Cancer treatment affects well-being and often prevents patients and caregivers from doing meaningful activities (27, 28).

These results open a window to non-pharmacological treatments to improve quality of life in children inside the hospital and consequently improve their treatment adherence.

## Data availability statement

The raw data supporting the conclusions of this article will be made available by the authors, without undue reservation.

## Ethics statement

The studies involving humans were approved by Comité de Ética del Instituto Nacional de Pediatrıáa (Approval number 2018-068/Approval date: November 29th, 2018). The studies were conducted in accordance with the local legislation and institutional requirements. Written informed consent for participation in this study was provided by the participants’ legal guardians/next of kin.

## Author contributions

LV: Writing – original draft, Writing – review & editing, Conceptualization, Investigation, Methodology, Project administration, Supervision. AG: Writing – review & editing, Writing – original draft, Conceptualization, Formal analysis, Investigation, Methodology. BS: Writing – original draft, Conceptualization, Funding acquisition, Investigation, Methodology. AE: Writing – original draft, Conceptualization, Funding acquisition, Investigation, Methodology. MM: Writing – original draft, Writing – review & editing, Formal analysis. CO: Writing – original draft, Conceptualization, Investigation, Methodology, Supervision. SC: Writing – original draft, Writing – review & editing, Conceptualization, Formal analysis, Investigation, Methodology, Supervision. LB: Writing – review & editing, Investigation. MZ: Writing – original draft, Writing – review & editing, Conceptualization, Data curation, Formal analysis, Investigation, Methodology, Supervision.
